# Novel tricyclic pyrrolo-quinolines as pharmacological correctors of the mutant CFTR chloride channel

**DOI:** 10.1038/s41598-023-34440-0

**Published:** 2023-05-10

**Authors:** Mario Renda, Marilia Barreca, Anna Borrelli, Virginia Spanò, Alessandra Montalbano, Maria Valeria Raimondi, Roberta Bivacqua, Ilaria Musante, Paolo Scudieri, Daniela Guidone, Martina Buccirossi, Michele Genovese, Arianna Venturini, Tiziano Bandiera, Paola Barraja, Luis J. V. Galietta

**Affiliations:** 1 Telethon Institute of Genetics and Medicine (TIGEM), Via Campi Flegrei 34, 80078 Pozzuoli, NA Italy; 2 Department of Biological, Chemical, and Pharmaceutical Sciences and Technologies (STEBICEF), University of Palermo, Via Archirafi 32, 90123 Palermo, Italy; 3 U.O.C. Genetica Medica, IRCCS Istituto Giannina Gaslini, Genova, Italy; 4 Department of Neurosciences, Rehabilitation, Ophthalmology, Genetics, Maternal and Child Health (DINOGMI), University of Genova, Genoa, Italy; 5 D3 PharmaChemistry, Istituto Italiano di Tecnologia (IIT), Genova, Italy; 6 Department of Translational Medical Sciences (DISMET), University of Naples “Federico II”, Naples, Italy

**Keywords:** Drug discovery, Physiology

## Abstract

F508del, the most frequent mutation in cystic fibrosis (CF), impairs the stability and folding of the CFTR chloride channel, thus resulting in intracellular retention and CFTR degradation. The F508del defect can be targeted with pharmacological correctors, such as VX-809 and VX-445, that stabilize CFTR and improve its trafficking to plasma membrane. Using a functional test to evaluate a panel of chemical compounds, we have identified tricyclic pyrrolo-quinolines as novel F508del correctors with high efficacy on primary airway epithelial cells from CF patients. The most effective compound, PP028, showed synergy when combined with VX-809 and VX-661 but not with VX-445. By testing the ability of correctors to stabilize CFTR fragments of different length, we found that VX-809 is effective on the amino-terminal portion of the protein that includes the first membrane-spanning domain (amino acids 1–387). Instead, PP028 and VX-445 only show a stabilizing effect when the second membrane-spanning domain is included (amino acids 1–1181). Our results indicate that tricyclic pyrrolo-quinolines are a novel class of CFTR correctors that, similarly to VX-445, interact with CFTR at a site different from that of VX-809. Tricyclic pirrolo-quinolines may represent novel CFTR correctors suitable for combinatorial pharmacological treatments to treat the basic defect in CF.

## Introduction

Cystic fibrosis (CF), one of the most frequent recessive genetic diseases, found primarily in Caucasians, is caused by mutations in the *CFTR* gene^1^. Such mutations disrupt the expression and/or function of the CFTR chloride channel, a plasma membrane protein present in many types of epithelial cells. Since CFTR is essential for transepithelial anion and water transport in different organs, its loss of function affects the lungs, pancreas, liver, sweat glands, and reproductive system^1–3^. The most severe manifestations of CF are in the respiratory system, with progressive development of obstructive pulmonary disease due to mucus hypersecretion and bacterial colonization^3^.

CFTR is a multi-domain membrane protein belonging to the superfamily of ATP-binding cassette (ABC) transporters^4,5^. Starting from the amino-terminus, CFTR is composed by a first membrane spanning domain (MSD1), a cytosolic nucleotide binding domain (NBD1), a regulatory (R) domain, a second membrane spanning domain (MSD2), and a second nucleotide binding domain (NBD2). MSD1 and MSD2, each one consisting of six membrane spanning helices, assemble together to form the pathway for bidirectional anion flux. NBD1 and NBD2 interact with each other forming two separate binding sites for ATP^4^. Binding and hydrolysis of ATP drive cycles of CFTR channel opening and closure. CFTR activity also requires cAMP-dependent phosphorylation of R domain.

Deletion of phenylalanine at position 508 (F508del) in the CFTR protein is the most frequent mutation^5^. F508del, which resides in NBD1, has a global negative effect on CFTR folding and stability, resulting in retention of the protein in the endoplasmic reticulum and early degradation^6–8^. F508del also causes an intrinsic defect in channel activity (gating defect). Indeed, if F508del-CFTR is forced to traffic to plasma membrane, by treating cells at low temperature or by overexpression, it shows a reduced time spent in the open chloride-conductive state compared to normal CFTR^9,10^.

The molecular defects caused by F508del and other *CFTR* mutations can be corrected by small molecules called correctors and potentiators^5,11,12^. Correctors, such as VX-809, VX-661, and VX-445 (a.k.a. lumacaftor, tezacaftor, and elexacaftor, respectively), target the misfolding and instability caused by F508del allowing the CFTR protein to reach the plasma membrane^13,14^. Potentiators, such as VX-770, overcome the channel-gating defect caused by F508del and other mutations^15,16^.

A modest improvement in clinical condition was initially obtained by treating patients homozygous for F508del with a drug combination, named Orkambi, including VX-809 and VX-770^17^. Later, this group of patients showed a similar extent of benefit with a drug combination, named Symdeko in US and Symkevi in Europe, in which VX-809 was replaced by VX-661^18^. A much better therapeutic outcome, particularly on patients with a single copy of F508del, was obtained with a triple drug combination, named Trikafta in US and Kaftrio in Europe, that includes VX-445 in addition to VX-661 and VX-770^14^. The inclusion of a second type of corrector is needed because the mistrafficking caused by F508del cannot be overcome by a single agent. Actually, F508del causes multiple defects to CFTR protein, including intrinsic instability of the nucleotide binding domain 1 (NBD1), where F508del is localized, and altered interaction of NBD1 with other CFTR domains^19,20^. In this respect, it has been shown that there are at least three classes of correctors. C1 correctors, in particular VX-809 and VX-661, act by interacting with TMD1^21^. C2 correctors, like the bithiazole corr-4a and compound 3151, act on the second half of the CFTR protein^19^. C3 correctors, which include chemical chaperones like glycerol and the small molecules 4172 and VX-445, appear to improve the stability of NBD1^19,22,23^. Identification of novel correctors will help in the design of combinatorial treatments targeting F508del and other CF mutations with similar defects.

The bithiazole corr-4a, identified by high-through screening of maximal diversity chemical library, was one of the first examples of F508del-CFTR correctors^24^. Corr-4a showed potency in the micromolar range and modest efficacy in native airway epithelial cells from CF patients. To improve corrector activity, various chemical modification of the basic bithiazole scaffold have been attempted. One solution was to generate constrained bithiazoles bearing a central ring ranging from 5 to 8 carbon atoms^25,26^. This modification, i.e. the synthesis of molecules with three fused rings, improved by 50% the maximal efficacy compared to free-rotating bithiazoles. Interestingly, tricyclic molecules have been shown to affect CFTR synthesis and/or function. Psoralens, belonging to the chemical family of linear furocumarins, were found to potentiate CFTR channel activity^27,28^. Furthemore, 4,6,4’-trimethylangelicin (TMA), an angular furocumarin, is a compound with both potentiator and corrector activity^29^. In the present study, we aimed at further exploring the corrector activity of tricyclic compounds by synthesizing and functionally testing a focused chemical library of nearly 200 compounds. After identification of an active hit, further chemical optimization led to compounds with greatly improved activity and high synergy with C1 correctors.

## Results

A set of 200 compounds having tricyclic structure were synthesized as heteroanalogues of angular furocumarins. These compounds, belonging to different chemical classes, consisted of pyrano- and thiopyrano-indoles; pyrrolo-thiopyrano pyridines; pyrrolo- and pyrazolo-quinolones; pyrrolo-quinazolines; pyrrolo-cyclohepta pyridines, pyrimidines and thiazole; thiazolo-, oxazolo- and triazolo-naphthyridines; thiazolo-indoles and isoindoles. This last class included 78 compounds, among which 21 were specifically prepared as analogues of constrained bithiazoles^30^.

**Figure 1:**
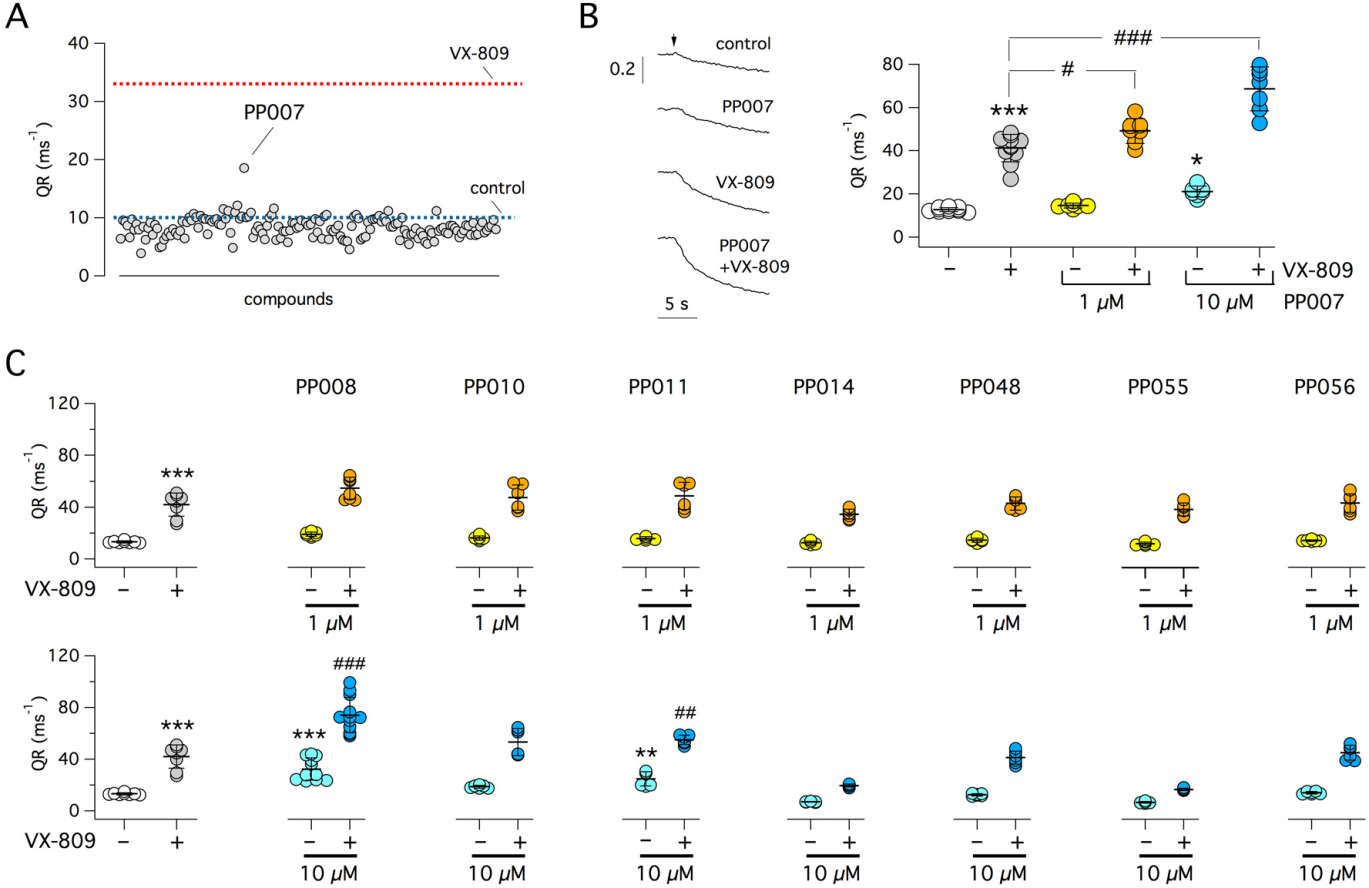
Identification of PP compounds as CFTR correctors. (**A**) Screening of a focused chemical library. The graph shows results obtained with the HS-YFP assay on CFBE41o- cells expressing F508del-CFTR and treated with test compounds (10 µM) for 24 h. Each circle reports CFTR activity (quenching rate, QR) for a single compound. Dotted lines show average activity of negative (vehicle) and positive (VX-809, 1 µM) controls. PP007 was active as a corrector. (**B**) Representative HS-YFP assay traces (left) and summary of data (right) revealing synergy of PP007 with VX-809. Quenching of HS-YFP was induced by extracellular addition of I^–^rich solution (arrow). *, *p* < 0.05; ***, *p* < 0.001; vs. vehicle. #, *p* < 0.05; ###, *p* < 0.001; vs. VX-809 alone (ANOVA with Tukey’s post-hoc test). (**C**) Activity data (HS-YFP assay) for indicated compounds, tested at 1 and 10 µM plus/minus VX-809 (1 µM). PP008 and PP011 are effective correctors. **, *p* < 0.01; ***, *p* < 0.001; vs. vehicle. ##, *p* < 0.01; ###, *p* < 0.001; vs. VX-809 alone (ANOVA with Tukey’s post-hoc test).

**Figure 2:**
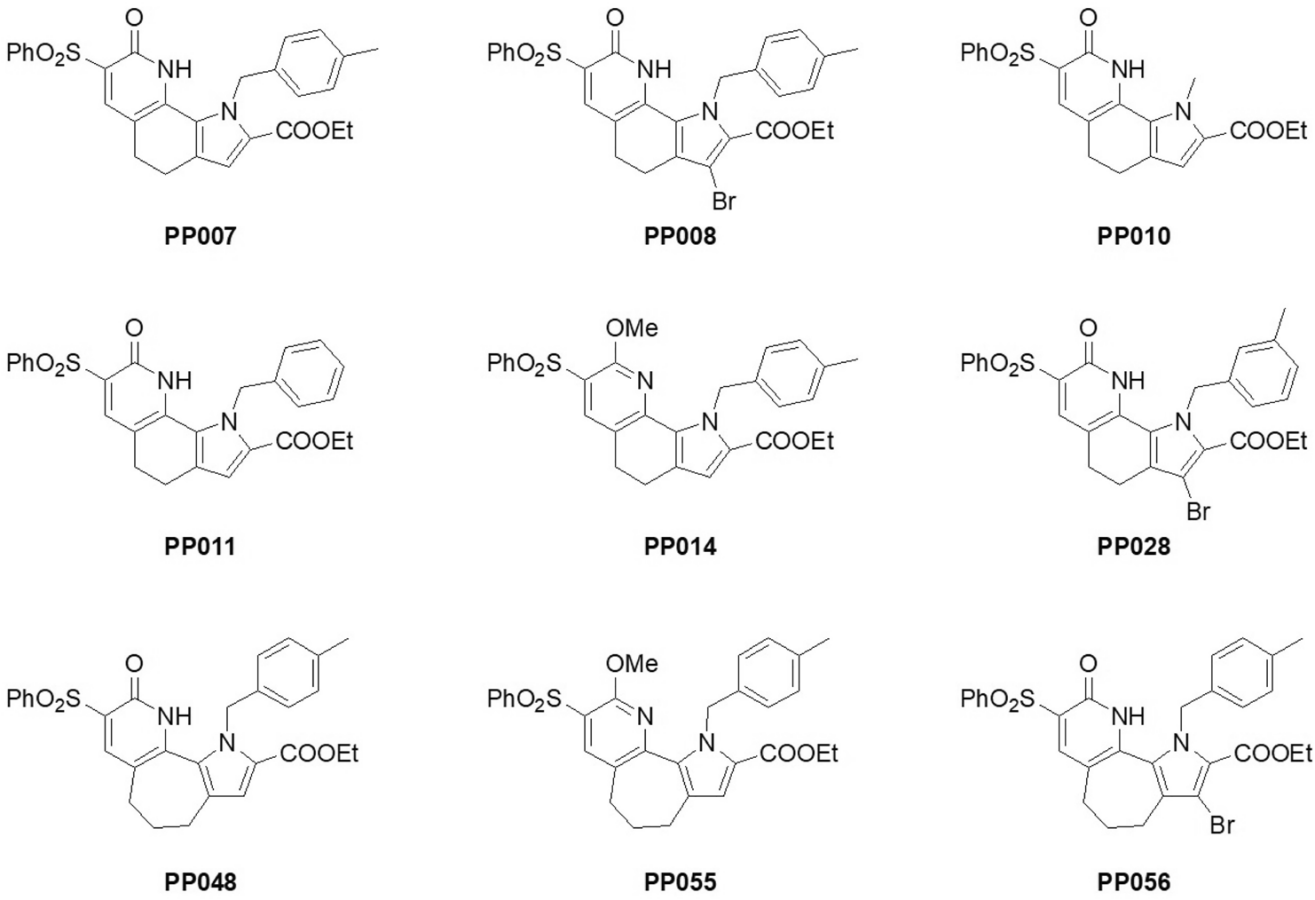
Chemical structures of PP compounds.

**Figure 3:**
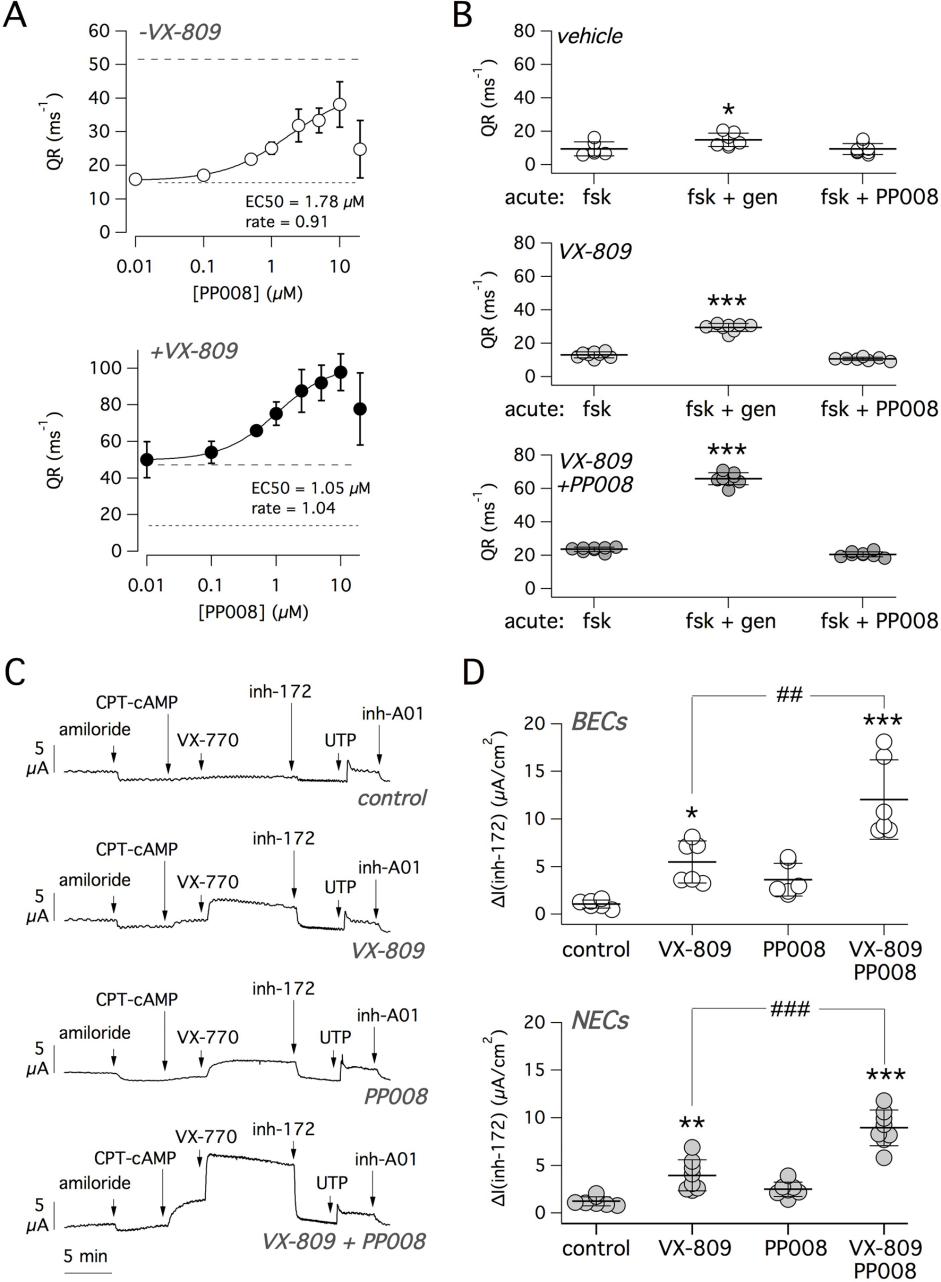
Characterization of PP008 corrector. (**A**) Dose–response relationship for PP008 as F508del-CFTR corrector in the absence (top) and presence (bottom) of VX-809 (1 µM). Data (mean ± SD) were obtained with the HS-YFP data on CFBE41o- cells. Dotted and dashed lines indicate average activity of vehicle and VX-809, respectively. Data are fitted with a Hill function. Values of EC_50_ and rate (Hill coefficient) are shown. (**B**) Evaluation of PP008 as a potentiator. Graphs show F508del-CFTR activity in CFBE41o- cells treated for 24 h with vehicle (top), 1 µM VX-809 (middle), 10 µM PP008 plus 1 µM VX-809 (bottom). After treatment and before the HS-YFP assay, cells were acutely stimulated with 20 µM forskolin (fsk), forskolin plus 50 µM genistein (gen), or forskolin plus 10 µM PP008. *, *p* < 0.05; ***, *p* < 0.001 vs. forskolin alone (ANOVA with Tukey’s post-hoc test). (**C**) Representative short-circuit current recordings from bronchial epithelia of a F508del/F508del patient. Epithelia were treated for 24 h with vehicle, 1 µM VX-809, 10 µM PP008, or both compounds together. Short-circuit current was recorded during sequential addition of amiloride (10 µM), CPT-cAMP (100 µM), VX-770 (1 µM), CFTR_inh_-172 (inh-172, 10 µM), UTP (100 µM), CaCC_inh_-A01 (inh-A01, 50 µM). (**D**) Summary of short-circuit current experiments from bronchial epithelial cells (BECs) and nasal epithelial cells (NECs). Data report the amplitude of CFTR_inh_-172 effect in epithelia treated with indicated conditions. *, *p* < 0.05; **, *p* < 0.01; ***, *p* < 0.001; vs. vehicle (control). ##, *p* < 0.01; ###, *p* < 0.001; vs. VX-809 (ANOVA with Tukey’s post-hoc test).

All compounds were tested as correctors on CFBE41o- cells expressing F508del-CFTR and the halide-sensitive yellow fluorescent protein (HS-YFP). Cells were incubated for 24 h with compounds at 10 µM (Fig. 1A). As positive control, cells were separately incubated with the corrector VX-809 (1 µM). After treatment, compounds were removed and cells were acutely stimulated for 20–30 min with 20 µM forskolin (to increase cytosolic cAMP) plus 50 µM genistein (as a potentiator to further enhance CFTR channel opening). F508del-CFTR activity in the plasma membrane was evaluated by measuring the rate of HS-YFP quenching elicited by extracellular addition of an iodide-rich saline solution. Rescue of F508del-CFTR results in enhanced iodide influx and hence accelerated fluorescence quenching. Only one compound, later labeled as PP007, elicited a relatively modest increase in F508del-CFTR function corresponding to ~ 30% of VX-809 effect (Figs. 1A and 2). PP007 was retested at 1 and 10 µM in the presence and absence of 1 µM VX-809 (Fig. 1B). Activity was found at 10 but not at 1 µM. Importantly, the combination of PP007 (1 and 10 µM) with VX-809 improved F508del-CFTR function with respect to VX-809 alone. In particular, a near doubling of VX-809 rescue was observed when 10 µM PP007 was included. This interesting result prompted the synthesis and evaluation of a small set of PP007 analogs. A few compounds, having modifications at different positions of the scaffold, were synthesized and tested on CFBE41o- cells with the HS-YFP assay (Figs. 1C and 2). In particular, a bromine atom was introduced at position 3 (PP008), the *p*-methylbenzyl moiety at the pyrrole nitrogen was replaced by a methyl (PP010) or an unsubstituted benzyl (PP011) group, and the carbonyl of the pyridone moiety replaced by a methoxy group (PP014). Compounds PP008 and PP011 showed significant activity. Importantly, PP008 emerged as a very effective corrector able to markedly synergize with VX-809. We tested PP008 at multiple concentrations in the range 0.01–20 µM (Fig. 3A, top). A maximal rescue, corresponding to nearly 70% of VX-809 effect, was achieved at 10 µM with an EC_50_ of ~ 1.78 µM. Interestingly, when tested in the presence of VX-809, the EC_50_ was decreased to 1.05 µM (Fig. 3A, bottom). At 20 µM, PP008 caused a decrease in function with respect to 10 µM, either with and without VX-809 (Fig. 3A).

We wondered whether PP008 has an effect on CFTR channel gating as a potentiator. Therefore, we incubated cells for 24 h with vehicle, with VX-809, or with VX-809 plus PP008. Then cells were acutely stimulated with forskolin, forskolin plus genistein, or forskolin plus PP008. In contrast to genistein, PP008 was unable to potentiate F508del-CFTR activity (Fig. 3B), irrespective of the type of corrector treatment.

The interesting properties of PP008 as a corrector encouraged us to test it in native airway epithelial cells. Figure 3C shows representative short-circuit current recordings on cultured bronchial epithelia from a F508del/F508del patient. During these recordings, F508del-CFTR function was maximally stimulated with CPT-cAMP followed by the VX-770 potentiator and then inhibited with the selective CFTR inhibitor-172 (inh-172)^31^. The net chloride secretion dependent on F508del-CFTR activity, and hence the efficacy of rescue maneuvers, was estimated from the amplitude of inhibitor effect. Figure 3D shows the results obtained from multiple preparations of bronchial and nasal epithelial cells of F508del/F508del patients. Cells treated with the combination of PP008 (10 µM) plus VX-809 showed significantly enhanced F508del-CFTR function with respect to cells treated with VX-809 alone. As shown in Fig. 3C, UTP was added at the end of experiments to elicit Ca^2+^-dependent Cl^-^ secretion, a process that involves activation of the TMEM16A Cl^-^ channel^32^. The size of the response to UTP was not affected by treatment with correctors, either PP008 or VX-809.

Given the very positive results obtained with PP008, a campaign of chemical synthesis and functional evaluation was undertaken to explore the structure–activity relationships (SAR) within the chemical class in order to find correctors with improved potency and efficacy. A series of modifications were introduced into the scaffold of PP008. Replacement of the *p*-methylbenzyl moiety of PP008 with a *m*-methylbenzyl group (PP028; Fig. 2) resulted in a substantial improvement in potency and efficacy. The enlargement of the central ring to seven carbon atoms (PP048, PP055, and PP056; Fig. 2) led to loss of activity. A number of compounds were prepared bearing substituted benzyl moieties at the pyrrole nitrogen; in many cases, these analogues maintained good activity compared with PP028. The SAR studies showed that crucial structural features for maintaining a good corrector activity are the presence of the pyridin-2-one moiety bearing a benzenesulfonyl substituent and of a carboxyl ester group. Hydrolysis of the ester to the corresponding carboxylic acid or transformation into a carboxamide produced compounds devoid of activity.

**Figure 4:**
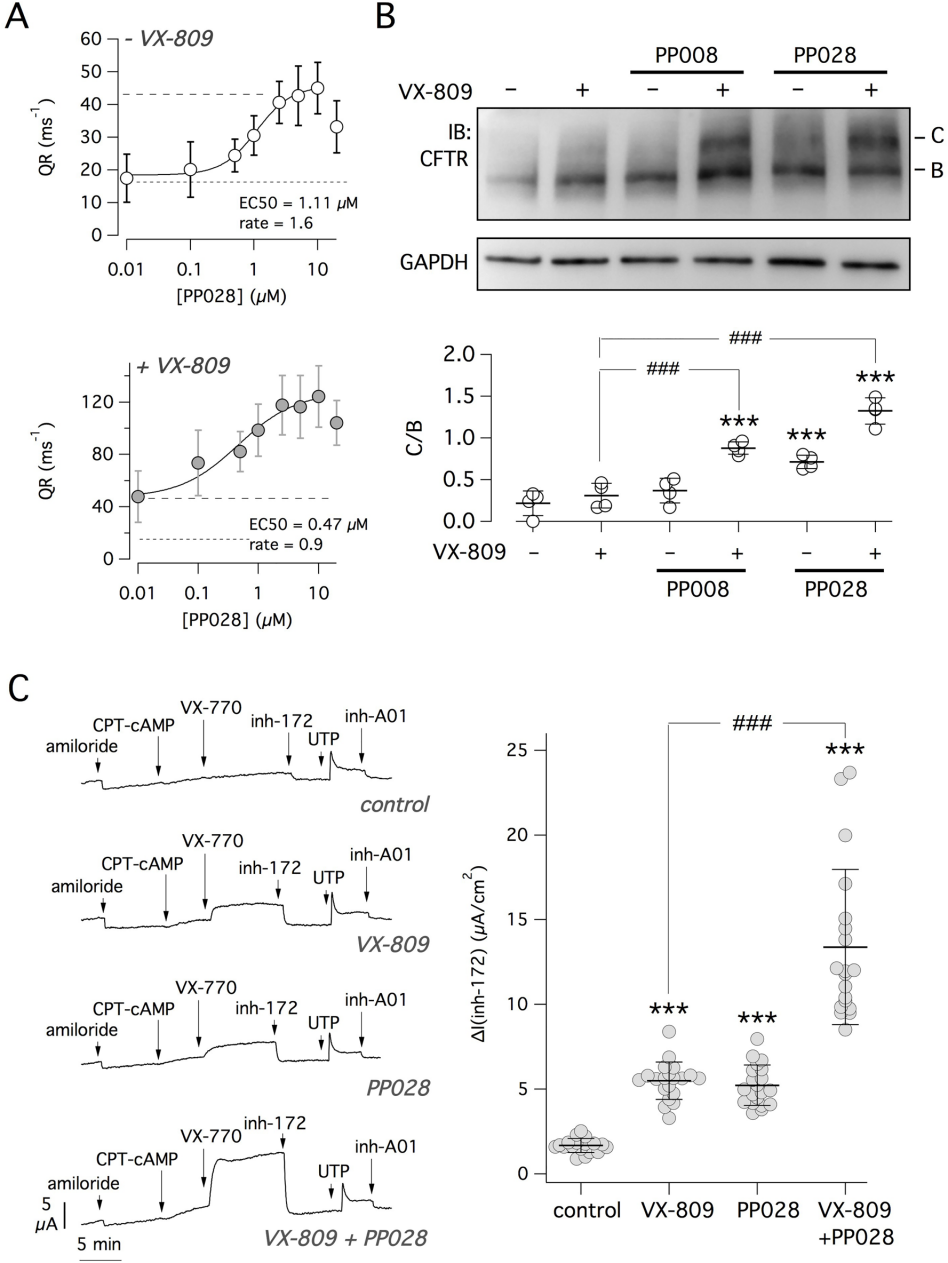
Characterization of PP028 corrector. (**A**) Dose–response relationship for PP028 as F508del-CFTR corrector in the absence (top) and presence (bottom) of VX-809 (1 µM). Data (mean ± SD) were obtained with the HS-YFP data on CFBE41o- cells. Dotted and dashed lines indicate average activity of vehicle and VX-809, respectively. Data are fitted with a Hill function. Values of EC_50_ and rate (Hill coefficient) are shown. (**B**) Top: detection of F508del-CFTR (C and B bands) and GAPDH in cell lysates by immunoblot. CFBE41o- cells were treated with vehicle, VX-809 (1 µM), and PP008 or PP028 (10 µM), plus/minus VX-809. Bottom: densitometric analysis of F508del-CFTR protein maturation. Data report band C/band B ratio, following normalization for GAPDH. ***, *p* < 0.001; vs. vehicle. ###, *p* < 0.001 vs. VX-809 (ANOVA with Tukey’s post-hoc test). (**C**) Representative short-circuit current recordings (left) and summary of data (right) from experiments on bronchial epithelia from F508del/F508del patients. Epithelia were treated for 24 h with vehicle, 1 µM VX-809, 10 µM PP028, or VX-809 plus PP028. Short-circuit current was recorded during sequential addition of amiloride (10 µM), CPT-cAMP (100 µM), VX-770 (1 µM), CFTR_inh_-172 (inh-172, 10 µM), UTP (100 µM), CaCC_inh_-A01 (inh-A01, 50 µM). The scatter dot plot shows the amplitude of CFTR_inh_-172 effect. ***, *p* < 0.001 vs. control. ###, *p* < 0.001 vs. VX-809 (Kruskal–Wallis non parametric test).

We focused our subsequent validation and mechanistic studies on PP028 as one of the most effective correctors. In CFBE41o- cells, this compound increased anion transport in a dose-dependent way with an EC_50_ of 1.1 µM (Fig. 4A, top). At the maximally-effective PP028 concentration, F508del-CFTR function was equivalent to that of cells treated with VX-809 alone. In the presence of VX-809, the effect of PP028 was largely amplified (Fig. 4A, bottom). In particular, the synergy generated by the combination of the two correctors elicited a nearly three-fold increase in anion transport with respect to each compound alone. Interestingly, as already seen with PP008, the EC_50_ of PP028 was significantly decreased (from 1.1 to 0.5 µM) in the presence of VX-809.

To further validate the corrector activity of PP028, we evaluated its effect on F508del-CFTR protein. F508del-CFTR protein maturation and trafficking was investigated by determining its electrophoretic mobility (Fig. 4B). Under control conditions, F508del-CFTR mainly migrates as a single band of nearly 150 kDa, named band B, which corresponds to the immature partially-glycosylated form of the protein^19,20^. Treatment of cells with PP028, particularly in combination with VX-809, resulted in increased expression of a 180 kDa band, named as band C, which corresponds to the mature fully-glycosylated CFTR protein (Fig. 4B). PP008 was also effective but only in combination with VX-809 (Fig. 4B).

PP028 was tested in bronchial epithelial cells from F508del/F508del patients (Fig. 4C). The results confirmed our observations in the CFBE41o- cell line. The corrector was very effective in rescuing mutant CFTR function either alone and in combination with VX-809. Importantly, the effect of the two-corrector combination was an eight-fold increase in CFTR-dependent current with respect to vehicle-treated cells (Fig. 4C). As already observed for PP008, treatment of cells with PP028 did not alter Ca^2+^-dependent Cl^-^ secretion elicited by UTP.

**Figure 5:**
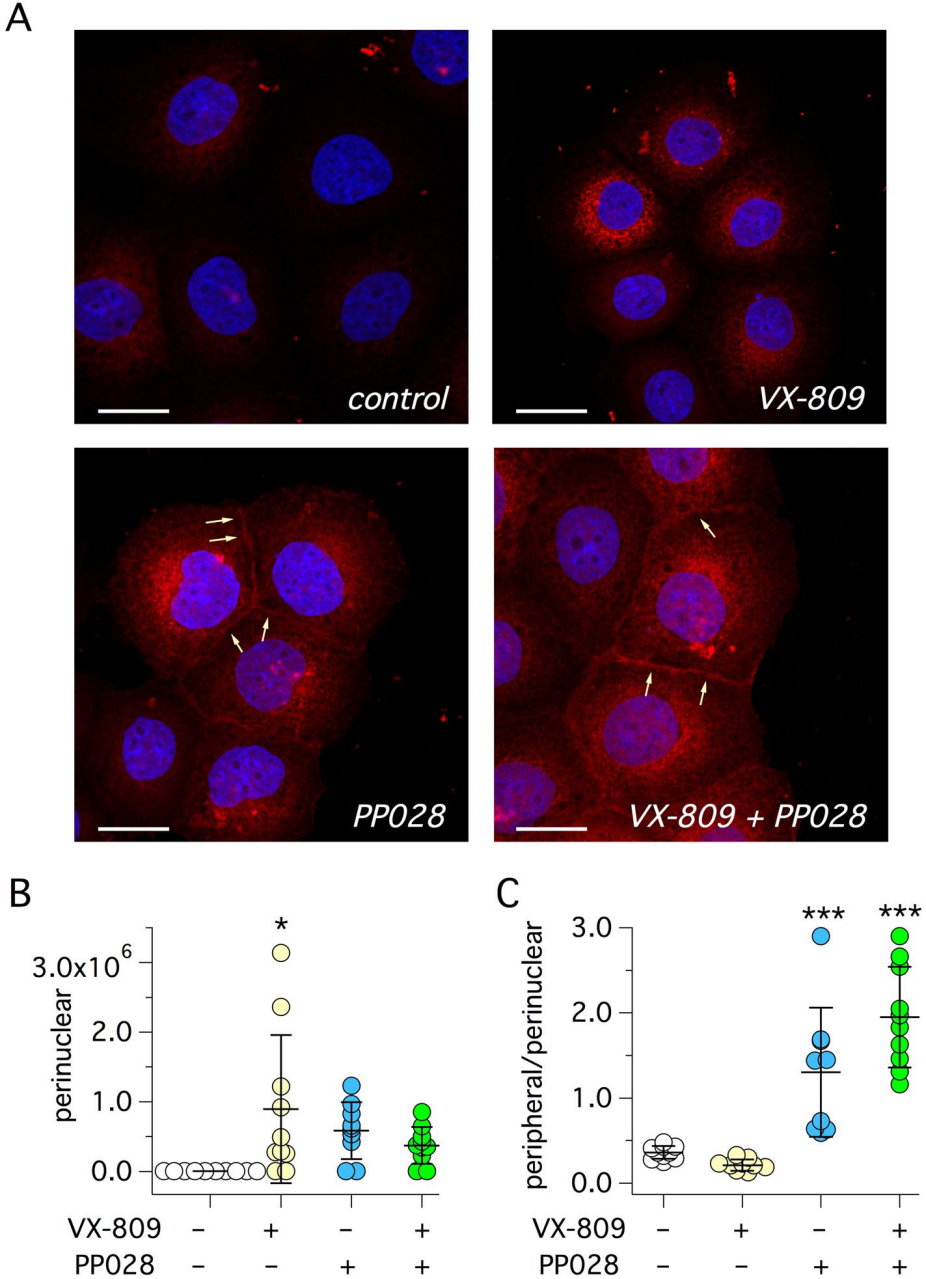
Detection of CFTR protein by immunofluorescence. (**A**) Representative images showing immunofluorescence detection of CFTR (red) in CFBE41o- cells expressing F508del-CFTR after 24 h treatment with vehicle (control), 1 µM VX-809, 10 µM PP028, and 1 µM VX-809 plus 10 µM PP028. Arrows show F508del-CFTR signal at the plasma membrane. Scale bar: 20 µm. (**B**) Intensity of CFTR signal in the perinuclear region of cells treated with indicated compounds. (**C**) Ratio of CFTR intensity signal in the peripheral and perinuclear regions. *, *p* < 0.05; ***, *p* < 0.001; vs. vehicle (ANOVA with Tukey’s post-hoc test).

We also investigated F508del-CFTR protein localization by immunofluorescence. We quantified CFTR signal in the perinuclear region and at the cell periphery. In vehicle-treated cells, the signal was weak and only localized in intracellular compartments (Fig. 5A). Treatment with VX-809 caused a significant increase in CFTR expression only in the perinuclear region (Fig. 5A,B). Instead, with PP028 treatment, perinuclear CFTR was not significantly increased but we noticed the appearance of a CFTR signal at the cell periphery, consistent with plasma membrane localization (Fig. 5A). Accordingly, the ratio of peripheral to perinuclear signal was significantly increased by PP028 (Fig. 5C). The peripheral signal was more evident by treating cells with the PP028/VX-809 combination (Fig. 5A).

**Figure 6:**
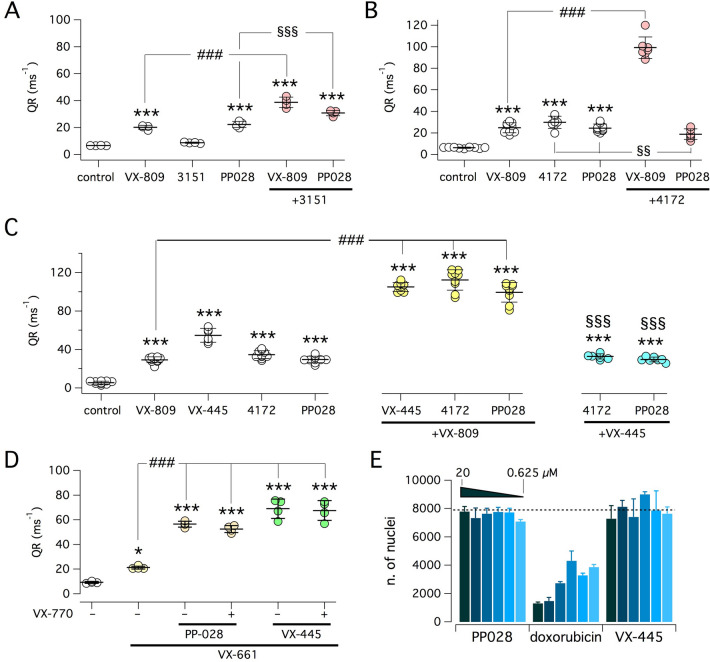
Functional evaluation of corrector combinations. Graphs show results obtained with the HS-YFP assay on CFBE41o- cells expressing F508del-CFTR. Cells were treated for 24 h with the following compounds as single treatments or as combinations: vehicle (DMSO), VX-809 (1 µM), 3151 (10 µM), PP028 (10 µM), 4172 (10 µM), VX-445 (5 µM), VX-661 (10 µM). (**A**) Effect of 3151 combinations with VX-809 or PP028. (**B**) Effect of 4172 combinations with VX-809 or PP028. (**C**) Effect of VX-445 and VX-809 combinations with 4172 or PP028. (**D**) Effect of PP028 and VX-445 with/without VX-661. Where indicated, VX-770 (1 µM) was also included in the 24 h treatment. In these experiments, VX-770 (1 µM) was used instead of genistein as potentiator. *, *p* < 0.05; ***, *p* < 0.001; vs. control. ###, *p* < 0.001 vs. VX-809 or VX-661. §§§, *p* < 0.001 vs. VX-445. §§, *p* < 0.01 vs. 4172 or PP028 (ANOVA with Tukey’s post-hoc test). (**E**) Citotoxicity test on CFBE41o- cells treated with indicated compounds at different concentrations (0.625, 1.2, 2.5, 5, 10, and 20 µM). Bars report the average number of nuclei (plus SD) per well.

As discussed in the Introduction section, correctors have been classified in at least three classes, depending on mechanism of action^22^. Combinations of compounds belonging to distinct classes generate additive/synergistic effects. Therefore, we combined PP028 with 3151 or with 4172, which have been classified as C2 and C3 correctors, respectively^22^. Compound 3151 appeared as a low efficacy corrector. It was ineffective if given alone and modestly enhanced the activity of VX-809 or PP028 when combined with these correctors (Fig. 6A). Instead, co-treatment of cells with VX-809 and 4172 generated a large synergic effect (Fig. 6B), comparable in size to that obtained with VX-809 plus PP028. Interestingly, combination of 4172 and PP028 revealed a sort of antagonism between the two compounds, with the activity resulting from the combination being smaller than that of either compound alone (Fig. 6B).

We also evaluated VX-445^14^, a second generation Vertex corrector that has been included in the triple combination of CFTR modulators, recently approved under the brand name Trikafta/Kaftrio^33^. Combination of VX-445 and VX-809 generated a high extent of synergy, comparable to that obtained with VX-809/4172 and VX-809/PP028 (Fig. 6C). No additivity/synergy was instead observed when VX-445 was combined with 4172 or PP028.

We tested the ability of PP028 to act synergistically with VX-661, which is the C1 corrector replacing VX-809 in the Symdeko/Symkevi and Trikafta/Kaftrio drug combinations. For these experiments, we also included chronic treatment with/without VX-770. Addition of PP028 significantly amplified the rescue by VX-661 (Fig. 6D). Inclusion of VX-770 did not change the effect of PP028/VX-661 combination. The results obtained with PP028/VX-661 were comparable with those obtained with VX-445/VX-661 (Fig. 6D).

We evaluated the possibility of cytotoxic effects elicited by PP028 treatment. CFBE41o- cells, plated at subconfluent density, were treated with PP028 at multiple concentrations in the range 0.625–20 µM. VX-445 and doxorubicin were also tested for comparison. After 24 h, cell number was quantified by counting the nuclei stained with NucBlue. We found no dose-dependent decrease in cell number with either PP028 or VX-445 (Fig. 6E) thus indicating lack of clear cytotoxic effects. In contrast, doxorubicin, as a control cytotoxic compound, markedly decreased cell number in a dose-dependent way (Fig. 6E).

**Figure 7:**
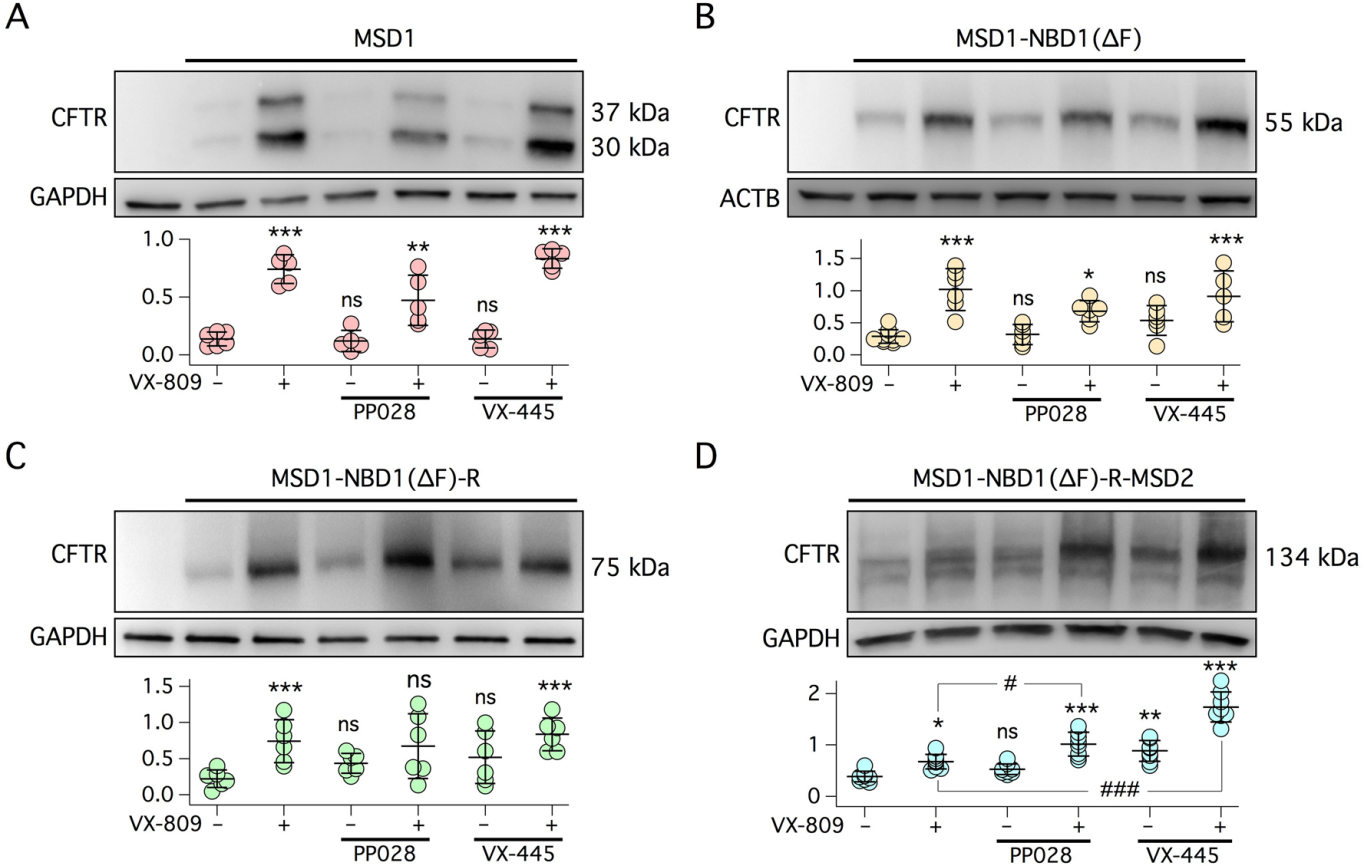
Stabilization of CFTR fragments by CFTR correctors. Each panel shows immunoblot analysis of cells transfected with MSD1 (**A**), MSD1-NBD1(∆F) (**B**), MSD1-NBD1(∆F)-R (**C**), and MSD1-NBD1(∆F)-R-MSD2 (**D**). Cells were incubated for 24 h with: vehicle, 1 µM VX-809, 10 µM PP028, or 5 µM VX-445, as single agents or as combinations. Scatter dot plots show densitometric analysis of CFTR fragments. For panel A, the upper band (37 kDa) was considered for analysis. Data report the ratio of CFTR fragment intensity to GAPDH or beta-actin (ACTB) band intensity. *, *p* < 0.05; **, *p* < 0.01; ***, *p* < 0.001; ns, not significant vs. vehicle. #, *p* < 0.05; ###, *p* < 0.001 vs. VX-809 (ANOVA with Tukey’s post-hoc test).

To investigate the site of action of PP028, we tested its ability to stabilize different fragments of CFTR protein, an assay that has been previously adopted to study other correctors^22,34,35^. PP028 activity was compared with that of VX-809 and VX-445. We first tested these three correctors on MSD1 fragment (amino acids: 1–387). By immunoblot, we detected two bands of 37 and 30 kDa, respectively (Fig. 7A). The upper band corresponds to the expected molecular size of the MSD1 fragment. The lower band may result from a proteolytic cleavage. We considered the 37 kDa band for densitometric analysis. As shown in Fig. 6A, VX-809, but not PP028 or VX-445, stabilized the MSD1 fragment, as evident from the increased band intensity. PP028 and VX-445 were not effective even if combined with VX-809 (Fig. 7A). We then tested the three correctors on MSD1-NBD1 fragment containing the F508del mutation (amino acids: 1–633). Expression of this construct resulted in a single band of the expected molecular size. MSD1-NBD1(∆F) was sensitive to VX-809 (Fig. 7B), but showed no significant stabilization by PP028 and VX-445, either alone or in combinations. Comparable results were also obtained with the fragment including the R domain, i.e. MSD1-NBD1(∆F)-R (amino acids: 1–823) (Fig. 7C). Finally, we tested correctors on the MSD1-NBD1(F508del)-R-MSD2 (amino acids: 1–1181; Fig. 7D). This construct, which includes most of CFTR domains except for NBD2 and the carboxy-terminus, was responsive to all three correctors. More precisely, PP028 was not effective by itself but significantly increased band intensity in combination with VX-809 compared to VX-809 alone (Fig. 7D). VX-445 was effective as a single treatment compared to vehicle and markedly enhanced fragment stability when combined to VX-809.

## Discussion

The identification of small molecules able to improve the maturation, trafficking, and gating of mutant CFTR is of high relevance to design pharmacological strategies to treat the basic defect in CF. In the present study, we have identified a novel class of correctors. The structure of angular furocumarins and constrained bithiazoles, encouraged the evaluation of several heteroanalogues of an in house library, which was the starting point of our chemical exploration. By screening our focused library, we found PP007 as the only active compound, with the interesting property of showing synergy when combined with VX-809, a typical C1 corrector^22^. Starting from PP007, we generated a series of analogues that led us first to PP008, a close analogue of hit PP007, having improved potency and efficacy on F508del-CFTR as corrector in cell lines and primary airway epithelial cells. Further modifications of the structure of PP008 allowed us to discover PP028, a derivative with a better activity profile than the parent compound. In the presence of VX-809, PP008 and PP028 do not only show enhanced efficacy but also an improvement in potency as indicated by the decrease in the EC_50_ value. These results suggest that VX-809 and PP compounds cooperate by acting on two different sites of CFTR protein. We combined PP028 with other types of correctors, including 3151, as a C2 corrector, and 4172 and VX-445, as C3 correctors^22,23^. We found synergy with 3151 but not with 4172 and VX-445. Actually, we found a sort of antagonism between PP028 and 4172, suggesting that the binding of one molecule negatively affects the binding and/or efficacy of the other molecule.

The lack of additivity/synergy between PP028, VX-445, and 4172 indicates that all of them belong to the same class of correctors. Previously, VX-445 and 4172 were classified as C3 correctors, with a possible mechanism of action involving binding to NBD1^22,23^. Therefore, according to our functional data, PP028 should also be a C3 corrector. Recently, VX-809 binding site was identified in MSD1^21^, in agreement with previous studies showing that VX-809 improves the stability of a CFTR fragment that only includes the first six transmembrane helices^34^. We have adopted the stabilization of CFTR fragments of different length as an assay to investigate the mechanism of action of PP028 in comparison with other CFTR correctors. As expected, VX-809 was the only compound that markedly stabilized MSD1. Surprisingly, VX-445 did not stabilize the MSD1-NBD1(∆F) fragment, despite previous indications, obtained with other types of assays, that VX-445 binds to NBD1. PP028 was also ineffective on the same fragment, as well as on MSD1-NBD1(∆F)-R. Instead, we found a stabilizing effect of PP028 and VX-445 in combination with VX-809, when MSD2 was included in the CFTR construct. VX-445 was also effective in the absence of VX-809. These results could imply that the binding site of C3 correctors is in the second transmembrane domain of CFTR. While finalizing our manuscript, a cryo-EM study was published showing that VX-445 indeed interacts with MSD2, more precisely with amino acid residues (Trp1098, Arg1102) of transmembrane helix 11^36^. Intriguingly, VX-445 does not bind to a well defined pocket inside CFTR but interacts with the surface of transmembrane region. Furthermore, a small portion of VX-445 also interacts with the *lasso* domain (Ser18, Arg21), which belongs to the CFTR amino-terminus^36^. Therefore, it appears that VX-445 forms a bridge between two distinct CFTR regions, a type of interaction that may be important for protein stabilization. It will be interesting to assess if PP compounds share the same binding site of VX-445.

In conclusion, our study has revealed a new family of CFTR correctors, showing a marked efficacy in primary airway epithelial cells from CF patients with F508del mutation. Considering that PP028, the best corrector of this family, was found within a relatively small set of analogues, we can postulate that further exploration of the chemical space around the PP scaffold, also supported by the structural information about the binding site of C3 correctors^36^, may lead to compounds with improved ability to rescue F508del and other CFTR mutants with trafficking defects. This is an important step since the potency and efficacy of PP compounds on mutant CFTR function and trafficking needs to be improved.

## Materials and methods

### Chemistry

Compounds shown in Fig. 2 were prepared according to our previously reported methods^37^.

### Cell culture

CFBE41o- cells, co-expressing F508del-CFTR and HS-YFP, and HEK293T cells were cultured in DMEM/Ham’s F12 (1:1) supplemented with 10% FBS, 2 mM L-glutamine, 100 U/ml penicillin, 100 µg/ml streptomycin (complete DMEM/F12 medium). The culture medium of CFBE41o- cells also contained puromycin (2 µg/ml) and G418 (0.75 mg/ml).

Human bronchial epithelial cells were obtained from the “Servizio Colture Primarie”, a service of Italian Foundation for Cystic Fibrosis. The collection of human bronchi for scientific purposes was approved by the relevant Ethical Committee (Comitato Etico Regione Liguria; registration number: ANTECER 042–09/07/2018 and CER 28/2020). The protocol for collection and culture of human bronchial epithelial cells was described in detail in a previous study^37^. Briefly, human bronchi, dissected from the lungs of CF patients undergoing lung transplant, were washed and incubated overnight at 4 °C in protease XIV solution. Epithelial cells were then detached by vigorously pipetting of bronchial lumen, pelleted by centrifugation, and dissociated by 5–10 min treatment with trypsin^38^. After neutralization of trypsin with complete DMEM/F12 medium, cells were centrifuged and resuspended in a serum-free medium. This medium contained a 1:1 mixture of LHC basal medium and RPMI 1640 (Thermo Fisher Scientific) plus hormones, growth factors, and other supplements as indicated previously^37^. Additionally, to promote the proliferation of basal stem cells^39^, the medium was supplemented with bone morphogenetic protein (BMP) antagonist (DMH-1, 1 µM; Tocris), transforming growth factor- β (TGF- β) antagonist (A 83–01, 1 µM; Tocris), and the rho-associated protein kinase 1 (ROCK1) inhibitor (Y-27632, 10 µM; Tocris). After 4–5 passages, cells were seeded (on Snapwell porous inserts (cc3801, Corning Costar; 500,000 cells per insert). After 24 h from seeding, the medium was switched to a 1:1 mixture of DMEM and Ham’s F12 plus 2% New Zealand Fetal Bovine Serum (Thermo Fisher Scientific) plus hormones, and supplements as previously indicated^37^. The medium was replaced daily on both sides of permeable supports for 7 days. Subsequently, the apical medium was totally removed and the medium was only maintained, with daily change, on the basolateral side (air–liquid interface condition). Cells were maintained under this condition for 2 weeks before carrying out the experiments.

### HS-YFP assay

CFBE41o- cells co-expressing F508del-CFTR and HS-YFP were plated at high density (35,000 cells/well) in black-wall clear-bottom 96-well microplates (cc3603 Corning). After 24 h, cells were treated for further 24 h with vehicle or compounds at various concentrations in complete culture medium. After final treatment, each well in the microplate was washed three times with 200 µl complete PBS. After washing, each well received 60 µl of an activating solution containing 20 µM forskolin and 50 µM genistein in PBS. In some experiments, genistein was omitted or replaced by PP008 (Fig. 3B) or replaced by 1 µM VX-770 (Fig. 6D). Cells were stimulated with this solution for 30 min. Then the microplate was transferred to a FLUOstar Omega microplate reader (BMG LABTECH) equipped with syringe pumps and excitation/emission filters optimized for Enhanced Yellow Fluorescent Protein, EYFP (ET500/20 × and ET535/30 m, respectively; Chroma Technology Corporation). The assay was done in “well mode”, which consisted in a continuous fluorescence reading of 14 s for each well (0.2 s sampling time, 10 flashes/sample). At 2 s from start, the syringe pump injected 165 µl of a modified PBS in which NaCl was replaced with NaI (170 µl/s flow rate). The fluorescence recording from each well was background substracted and then normalized for the initial value. The fluorescence decay resulting from I^-^ influx and HS-YFP quenching was fitted with an exponential function to derive the maximum quenching rate (dF/dt). This calculation was done using a procedure compiled in the Igor Pro software (WaveMetrics, Lake Oswego, OR, USA).

### Short-circuit current recordings

Snapwell inserts with differentiated bronchial epithelia were mounted in vertical chambers resembling Ussing system with internal fluid circulation (EM-CSYS-8, Physiologic Instruments). Both apical and basolateral compartments were filled with 5 ml of a bicarbonate-buffered solution containing (in mM): 126 NaCl, 0.38 KH_2_PO_4_, 2.13 K_2_HPO_4_, 1 CaCl_2_, 1 MgSO_4_, 24 NaHCO_3_, 10 glucose, and phenol red. Solution on both sides were bubbled with a gas mixture of 5% CO_2_-95% air and kept at 37 °C. The transepithelial voltage was clamped at 0 mV with an 8-channel voltage-clamp amplifier (VCC MC8, Physiologic Instruments, San Diego, CA, USA) connected to apical and basolateral compartments via Ag/AgCl electrodes and agar bridges (1 M KCl in 2% agar). The resulting short-circuited current from each channel was recorded on a personal computer with the Acquire & Analyse 2.3 software (Physiologic Instruments, San Diego, CA, USA).

### MSD1-NBD1(∆F)-R-MSD2 construct

The plasmid containing the coding sequence for the MSD1-NBD1(∆F)-R-MSD2 fragment (amino acid residues 1–1181) was obtained by site-direct mutagenesis (Quick Change II XL site-directed mutagenesis kit, Agilent Technologies) to introduce the stop codon (3546TAC → TAA or Y1182X) on the expression vector pcDNA3.1 containing the F508del-CFTR mutant. The correct sequence was verified by sequencing (Eurofins Genomics).

### Transfection of CFTR fragments

HEK293T cells were plated onto poly-L-lysine-coated 6-well microplates and grown to 60% confluence in DMEM/F12 complete medium supplemented with 2 mM L-glutamine. Cells were transiently transfected with plasmids containing the coding sequence for the different CFTR fragments. For this purpose, for each well we combined 5 µl of Lipofectamine 2000 (Thermo Fisher Scientific) and 2 µg of plasmid, each one previously dissolved in 250 µl of Opti-MEM. The transfection complexes were allowed to form for 1 h at room temperature in the dark before adding to the cells. After 24 h, cells were treated for further 24 h with fresh complete medium containing vehicle (DMSO), 1 µM VX-809, 10 μM PP028 with/without 1 μM VX-809, or 5 μM VX-445 with/without 1 μM VX-809.

### Detection of CFTR protein by immunofluorescence

CFBE41o- cells with expression of F508del-CFTR were seeded on µ-Slide 12-well removable chamber supports (Ibidi, Gräfelfing, Germany). Cells were treated for 24 h with vehicle or correctors in complete DMEM/F12 medium. Then, cells were fixed for 5 min at room temperature with 10% neutral buffered formalin (0501005Q, Bio-Optica, Milan, Italy). After three washings in PBS, cells were treated with a solution containing saponin as a permeabilizing agent and bovine serum albumin (BSA) as a blocking agent (0.05% saponin, 0.5% BSA, 50 mM NH_4_Cl, 0.02% NaNH_3_, in PBS) for 30 min at room temperature. Cells were then incubated overnight at 4 °C with rabbit IgG anti-CFTR (D6W6L, Cell Signaling Technology, Danvers, MA, USA) diluted 1:400 in blocking buffer. Following incubation with primary antibody, cells were incubated for 1 h in the dark with Alexa Fluor 555-conjugated secondary antibody (Thermo Fisher Scientific) diluted 1:200 in blocking buffer. After 3 washes in PBS, the silicone chamber was removed, and cells were mounted with Fluoroshield with DAPI (Sigma-Aldrich) to stain cell nuclei. Images were acquired with a laser scanning confocal microscope (Zeiss LSM 700, Oberkochen, Germany).

The mean CFTR fluorescence intensity signal was measured with Fiji (NIH) software in regions of interests (ROIs) placed in two different sites of the cell, perinuclear and peripheral. The perinuclear region was clearly visible because of nuclear DAPI staining. For the peripheral region, a localization compatible with CFTR trafficking to plasma membrane, we placed the ROI at the cell edge, identified in phase contrast images. Ten cells per condition were analyzed. For each cell, six measurements of perinuclear and peripheral fluorescence intensity were done and the results were averaged. Data are presented as perinuclear signal and ratio of peripheral/perinuclear signals.

### Immunoblot analysis of full length CFTR

CFBE41o- cells co-expressing F508del-CFTR and HS-YFP, plated on 6-well microplates, were treated for 24 h with correctors or vehicle and then lysed with a buffer containing 50 mM Tris–HCl (pH 8.0), 150 mM NaCl, 1% NP-40, 0.5% sodium deoxycholate, 0.1% SDS, 1 mM PMSF and complete protease inhibitor cocktail (Roche). Cell lysates were subjected to centrifugation at 14,500 rpm at 4 °C for 15 min. Protein concentration was determined with the BCA assay (Euroclone) following the manufacturer’s instructions and using bovine serum albumin as the standard. Equal amounts of proteins (20 μg) were separated on 4–15% mini protean TGX Precast gels (Bio-Rad laboratories, Hercules, CA, USA), transferred to a nitrocellulose membrane with Trans-Blot Turbo system (Bio-Rad Laboratories Inc., Hercules, CA, USA). The membrane was incubated overnight at 4 °C with an anti-CFTR rabbit primary antibody (D6W6L, Cell Signaling Technology, USA) diluted 1:1000 in Tris-buffered saline with 0.5% Tween-20 (TBST) plus 5% skimmed-milk. The membrane was then washed three times and incubated for 1 h with a polyclonal goat anti-rabbit antibody conjugated with horseradish peroxidase (Dako) diluted 1:10,000 in TBST plus 5% milk. Immunodetection was subsequently visualized by chemiluminescence using the SuperSignal West Femto Substrate (Thermo Fisher Scientific). For quantification, images were analyzed with the Image Quant TL software (GE Healthcare). For each lane, intensity of signal was measured in two regions of interest, corresponding to bands B and C (150 and 180 kDa). Data were normalized using the GAPDH loading control.

### Immunoblot analysis of CFTR fragments

Transiently-transfected HEK293T cells were treated for 24 h with correctors or vehicle and then lysed with a buffer containing 50 mM Tris–HCl (pH 7.4), 150 mM NaCl, 1% NP-40, 0.5% sodium deoxycholate, 0.1% SDS, 1 mM PMSF and complete protease inhibitor cocktail (Roche). Cell lysates were centrifuged at 17,000 rpm at 4 °C for 15 min. Total protein concentration of the supernatant was calculated using the BCA assay (Euroclone), following the manufacturer’s instructions. Equal amounts of protein (20 μg) were separated on 4–15% mini protean TGX Precast gels (Bio-Rad laboratories, Hercules, CA, USA) and transferred to nitrocellulose membranes with Trans-Blot Turbo system (Bio-Rad Laboratories Inc., Hercules, CA, USA). Membranes were blocked with 5% skimmed-milk in TBST for two hours at room temperature and then incubated over night at 4 °C in agitation with the primary antibody. For fragments MSD1, MSD1-NBD1(∆F), and MSD1-NBD1(∆F)-R we used mouse anti-CFTR monoclonal antibody, clone MM13-4 (Sigma-Aldrich). For the MSD1-NBD1(∆F)-R-MSD2 fragment, we used the rabbit anti-CFTR D6W6L antibody (Cell Signaling Technology). Primary antibodies were diluted 1:1000 in TBST plus 5% skimmed-milk. Membranes were then incubated for one hour at room temperature with anti-mouse or anti-rabbit secondary antibodies conjugated to horseradish peroxidase (Dako, Agilent) diluted 1:2000) in TBST plus 5% skimmed-milk.

After CFTR fragment detection, membranes were incubated with primary antibodies for GAPDH (for MSD1, MSD1-NBD1(∆F)-R, and MSD1-NBD1(∆F)-R-MSD2) or beta-actin (for MSD1-NBD1(∆F)). For GAPDH, we used the mouse MAB374 clone 65C antibody (Sigma-Aldrich). For beta-actin, we used the mouse A1978 antibody (Sigma-Aldrich). Both antibodies were diluted 1:10,000 and 1:2000, respectively, in TBST plus 5% skimmed-milk. For GAPDH detection, membranes were previously stripped with the Restore Western Blot Stripping Buffer (Thermo Fisher Scientific). The HRP-conjugated anti-mouse secondary antibody was used at dilution 1:20,000 for beta-actin and 1:50,000 for GAPDH. Proteins were visualized by chemiluminescence with the SuperSignal West Femto Substrate (Thermo Fisher Scientific). Images were obtained using the Molecular Imager UVITEC Cambridge System and subsequently analysed with Fiji software (National Institutes of Health, Bethesda, MD, USA). CFTR band intensities were analyzed as regions of interest and normalized against the GAPDH or beta-actin control.

### Cytotoxicity assay

CFBE41o- cells were seeded in 96-well plates (PhenoPlate, PerkinElmer) at a density of 20,000 cells per well. After 24 h, cells were treated for further 24 h with PP028, VX-445, and doxorubicin at various concentrations. Finally, cells were labeled with NucBlue (Invitrogen, Live Cell Stain ReadyProbes reagent). The images were acquired with the High Content Analysis System, OperettaCLS (PerkinElmer) and analyzed by Harmony Software (PerkinElmer).

### Statistical analysis and data visualization

Data are shown as scatter dot plots plus mean ± SD or as representative images. Each symbol in the scatter dot plots represents the result of an independent experiment. To assess statistically significant difference between groups of data, we first used the Kolmogorov–Smirnov test to assess normal distribution. For normally distributed data, we then used ANOVA followed by Tukey’s post hoc test. For data with non-normal distribution, we used Kruskal–Wallis with Dunn’s non parametric test. Statistical analysis was done with PRISM software (GraphPad). All graphs and figures were prepared with Igor Pro (WaveMetrics).

### Ethics approval and consent to participate

The collection and study of bronchial and nasal epithelial cells from human subjects was done according to the guidelines of the Declaration of Helsinki and approved by the “Comitato Etico Regione Liguria” (Registration Numbers: ANTECER, 042–09/07/2018 and CER 28/2020). Informed consent to participate in scientific studies and to publish data in research journal articles was also obtained from all subjects involved in the study.

## Supplementary Information

Supplementary Information.

## Data Availability

All data generated or analyzed during this study are included in the present article.
