# Supplementary material for: Novel tricyclic pyrrolo-quinolines as pharmacological correctors of the mutant CFTR chloride channel
Source: Sci Rep. 2023 May 10;13:7604. doi: 10.1038/s41598-023-34440-0 (PMC10172366; doi:10.1038/s41598-023-34440-0)
Supplement: Supplementary file 1 — Supplementary Information. [file 41598_2023_34440_MOESM1_ESM.pptx]

## Slide 1
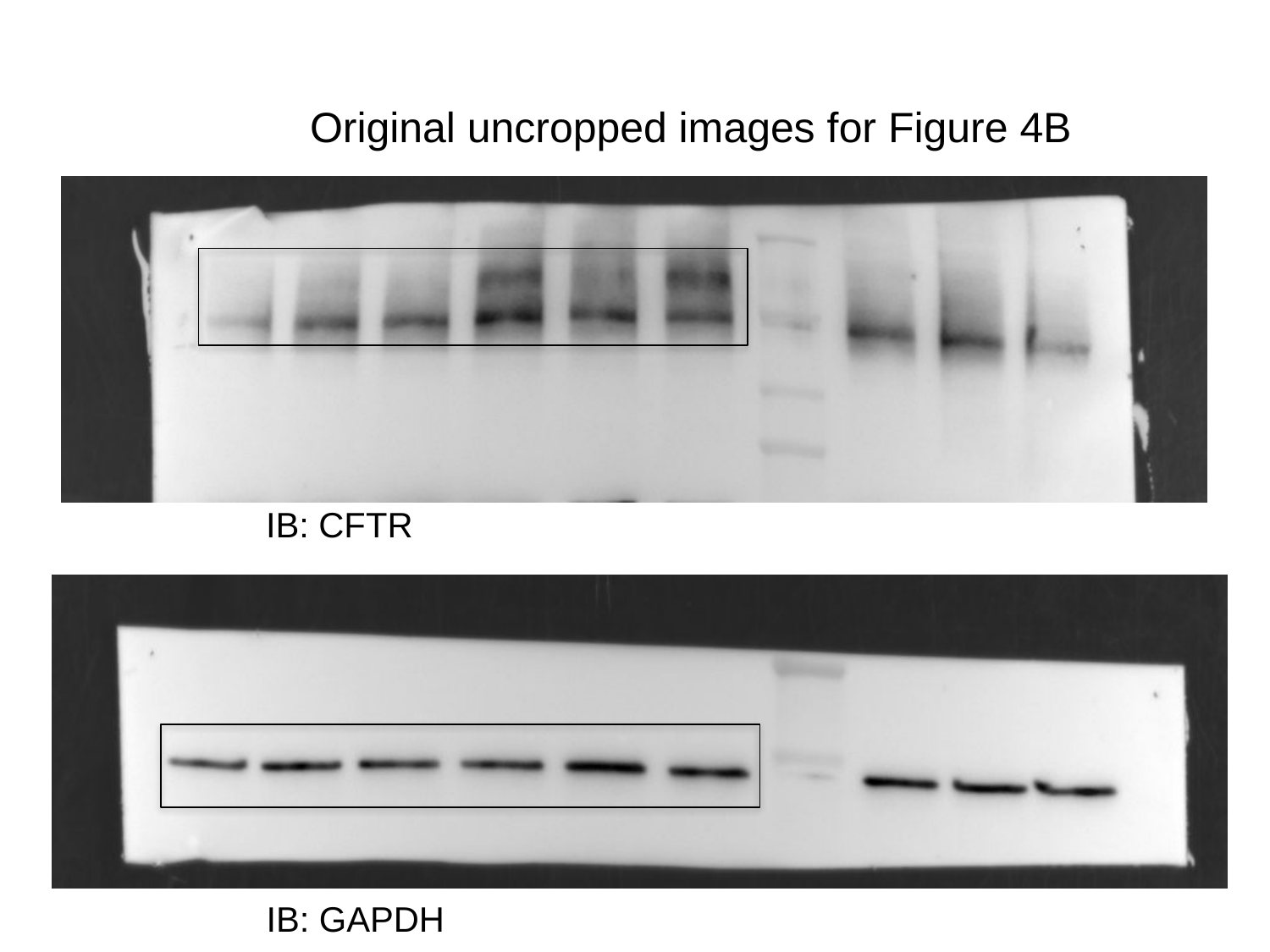

Original uncropped images for Figure 4B
IB: CFTR
IB: GAPDH

## Slide 2
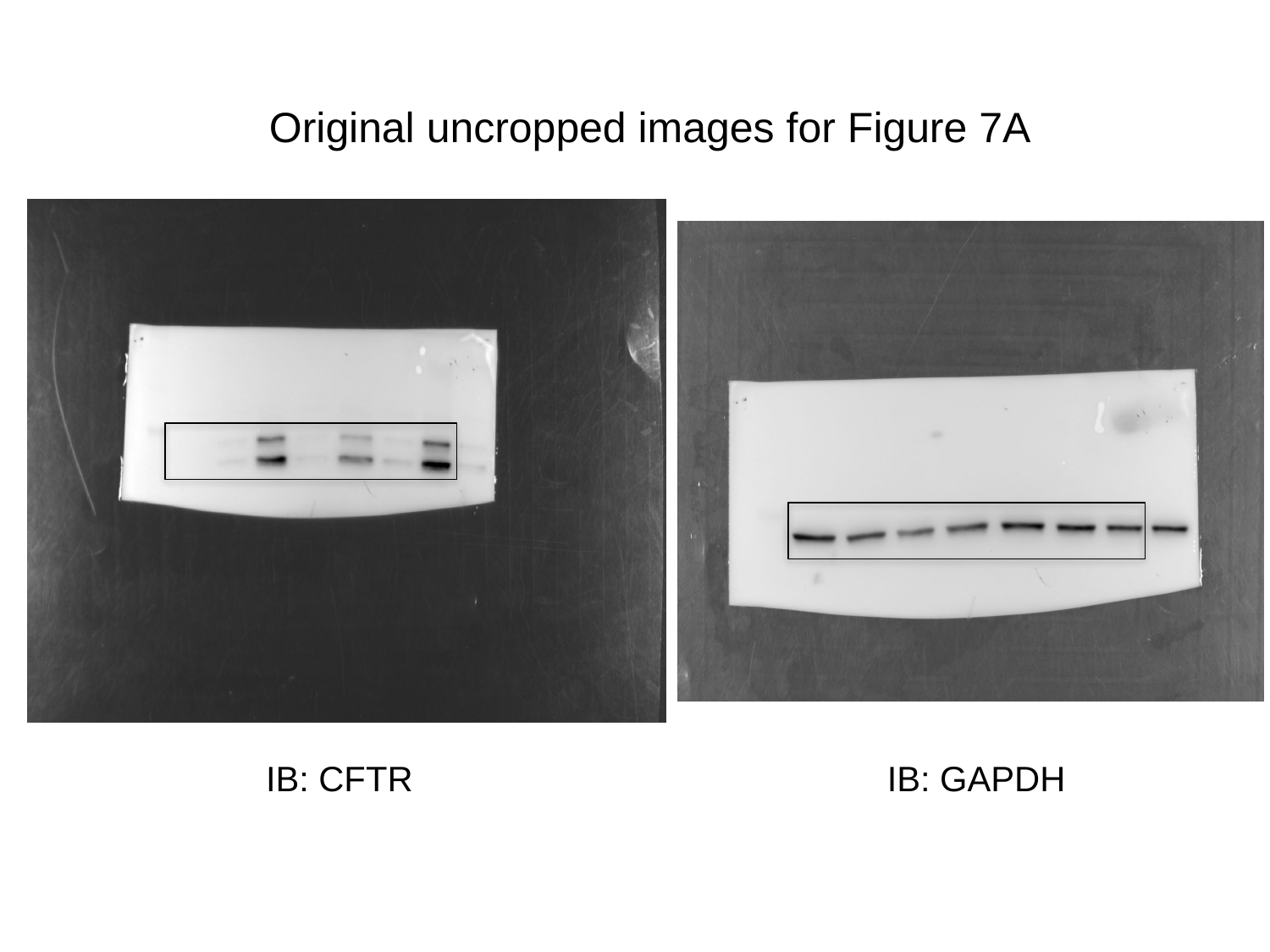

Original uncropped images for Figure 7A
IB: CFTR
IB: GAPDH

## Slide 3
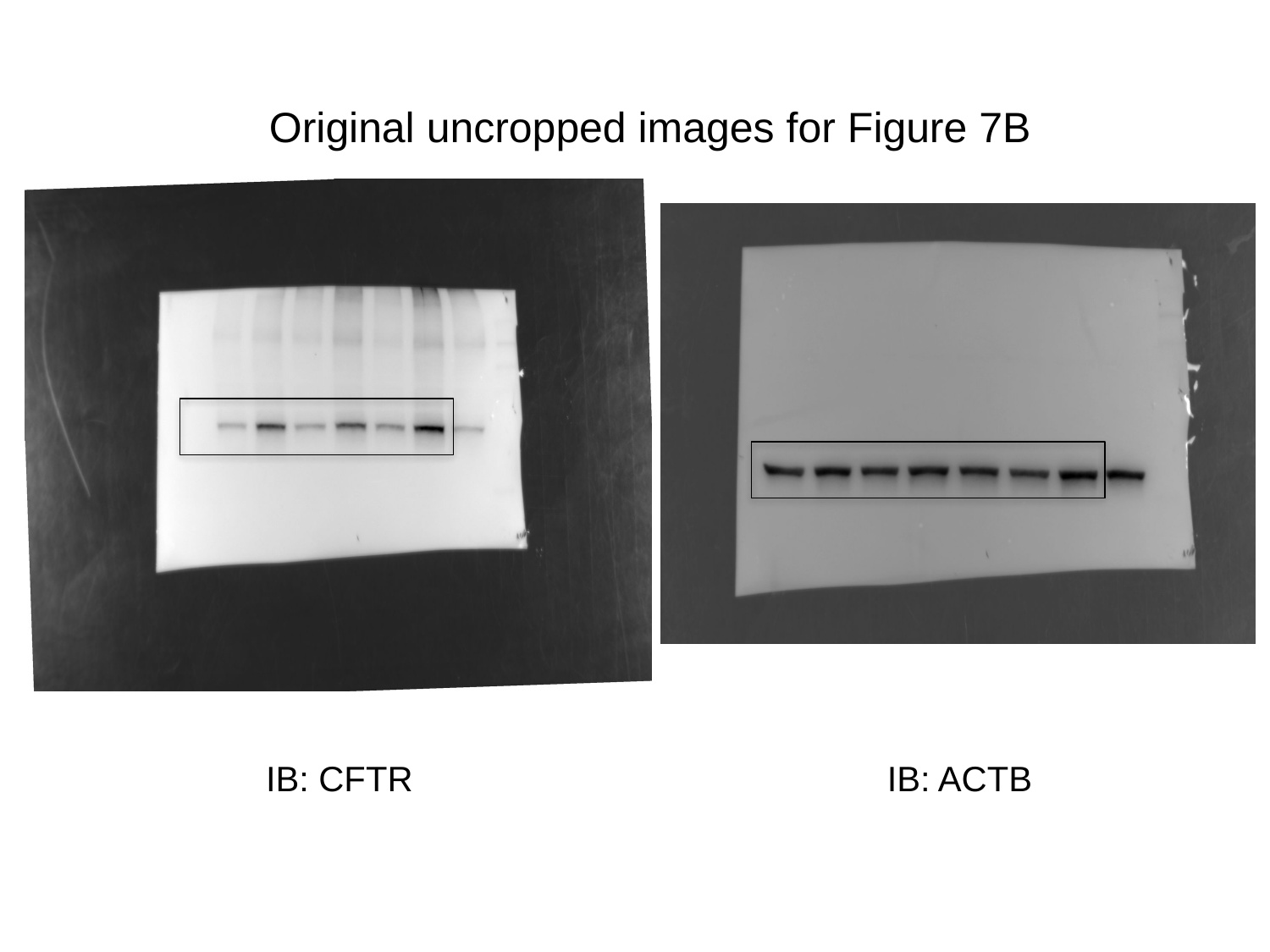

Original uncropped images for Figure 7B
IB: CFTR
IB: ACTB

## Slide 4
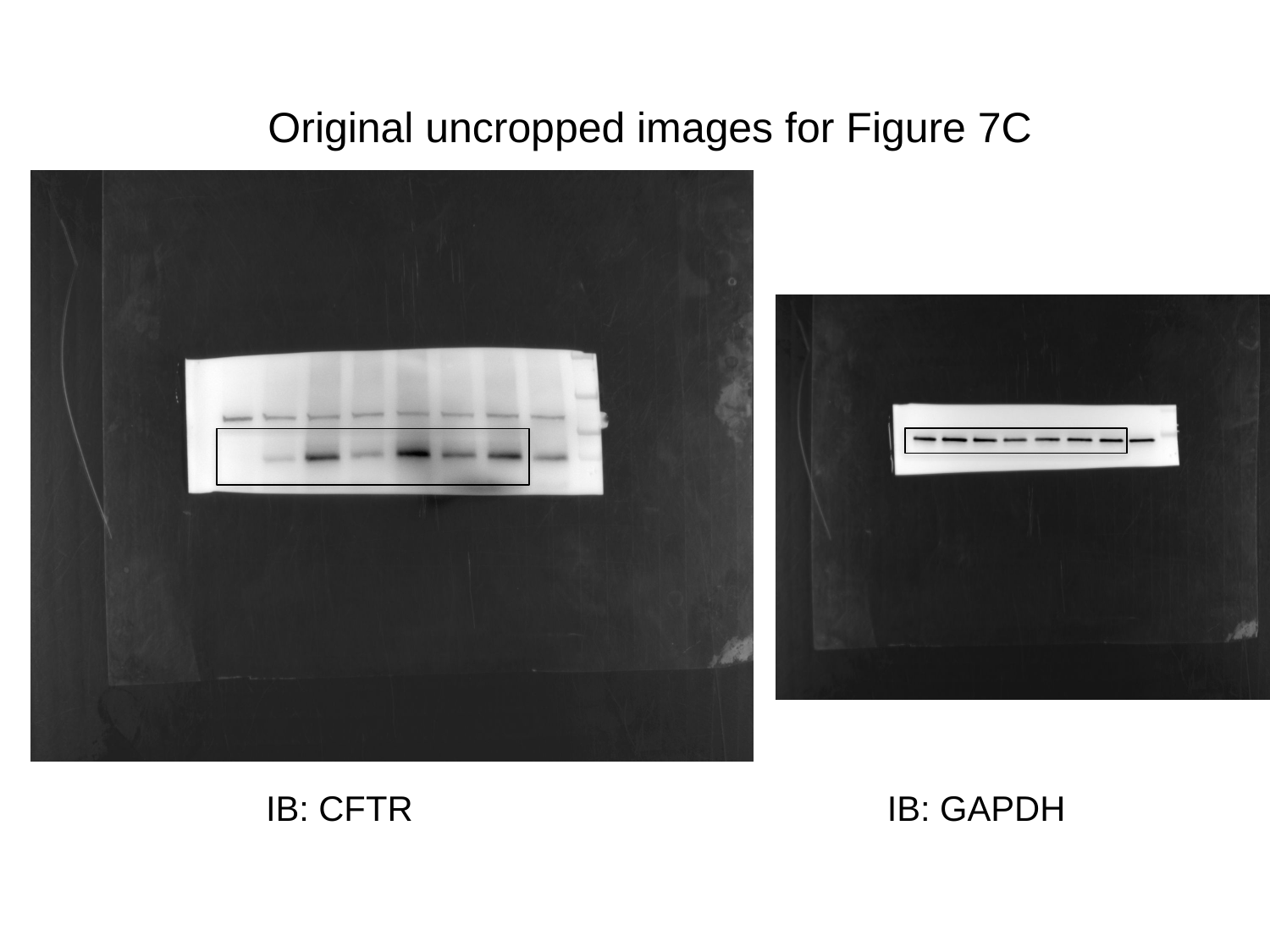

Original uncropped images for Figure 7C
IB: CFTR
IB: GAPDH

## Slide 5
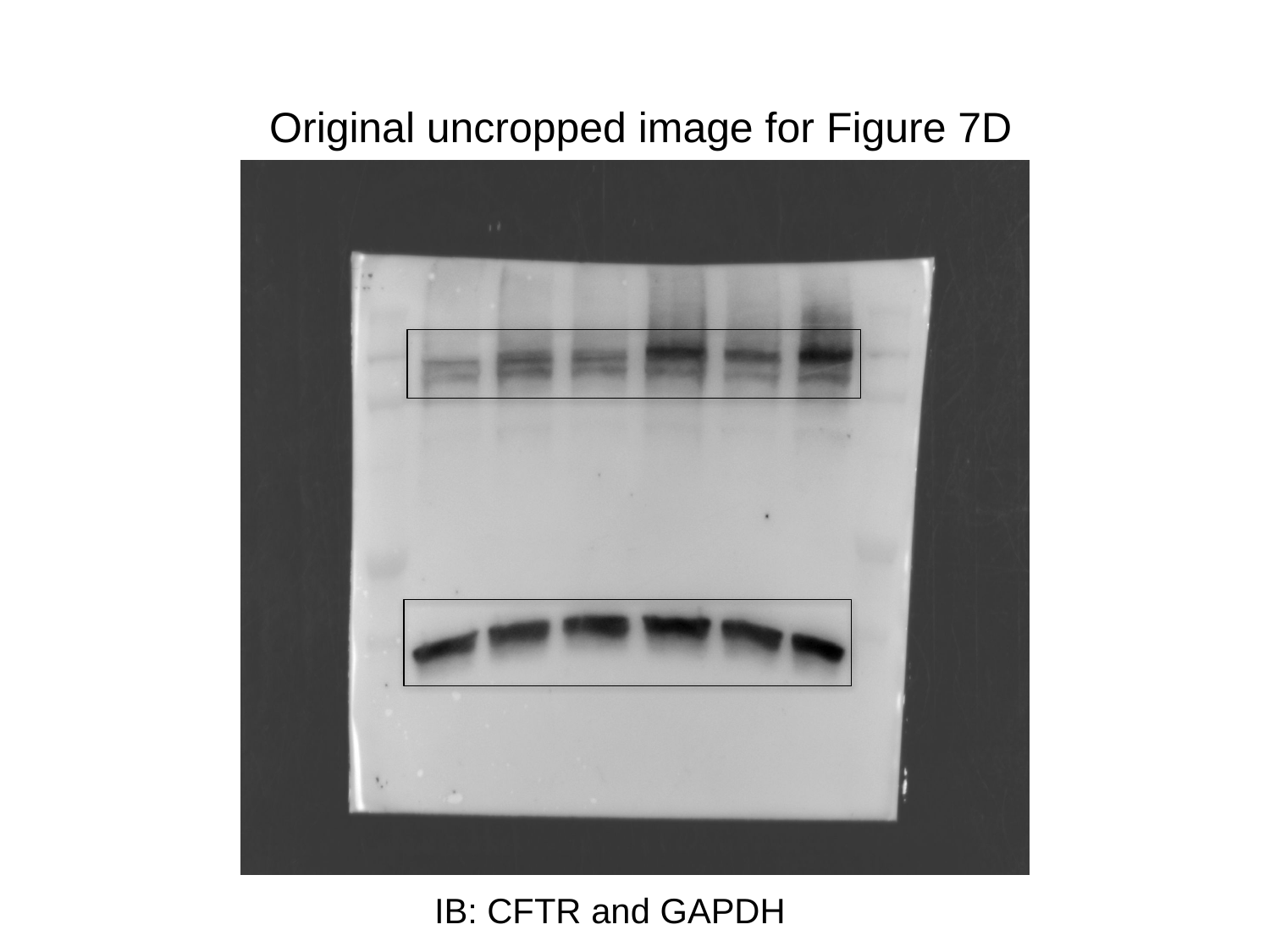

Original uncropped image for Figure 7D
IB: CFTR and GAPDH
